# Reticulate hypopigmentation of Dohi: a characteristic patterned pigmentary disorder

**DOI:** 10.11604/pamj.2026.53.76.50495

**Published:** 2026-02-10

**Authors:** Garima Gupta, Shweta Parwe

**Affiliations:** 1 Department of Panchkarma, Mahatma Gandhi Ayurveda College Hospital and Research Centre, Salod (Hirapur), Datta Meghe Institute of Higher Education and Research, Wardha, Maharashtra, India

**Keywords:** Reticulate hypopigmentation of Dohi, dyschromatosis, genodermatosis, hypopigmented macules

## Image in medicine

A 23-year-old male presented with asymptomatic hypopigmented lesions over the dorsal aspects of the hands, feet, and neck, noticed since early childhood. The lesions were non-progressive and symmetrically distributed. Cutaneous examination revealed multiple, well-defined, reticulate hypopigmented macules over the acral regions without associated erythema, scaling, or atrophy. No involvement of the trunk, face, hair, nails, or mucosa was noted. There was no significant past medical history. The diagnostic approach was based on the characteristic acral distribution, reticulate pattern of hypopigmentation, early onset, and absence of inflammatory changes, which are typical features of reticulate hypopigmentation of Dohi, a rare inherited pigmentary disorder. The patient was counselled regarding the benign nature of the condition, and reassurance was provided. No active medical treatment was initiated. At short-term follow-up, the lesions remained stable without progression.

**Figure 1:**
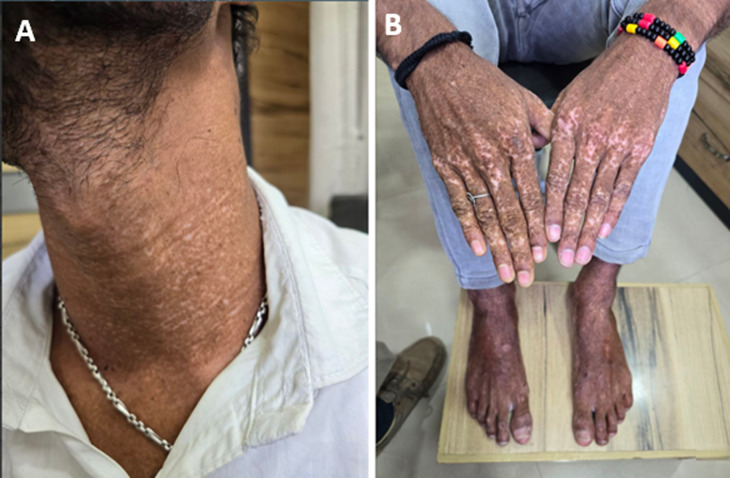
A) reticulate hypopigmented macules over the lateral neck; B) symmetrical reticulate hypopigmented macules over the dorsal hands and feet

